# Immune microenvironment and immunotherapy in hepatocellular carcinoma: mechanisms and advances

**DOI:** 10.3389/fimmu.2025.1581098

**Published:** 2025-04-02

**Authors:** Dong Xie, Yang Liu, Fangbiao Xu, Zhibo Dang, Mengge Li, Qinsheng Zhang, Zhongqin Dang

**Affiliations:** ^1^ Diagnosis and Treatment Center for Digestive Diseases of Henan Province Hospital of Traditional Chinese Medicine, Zhengzhou, China; ^2^ College of Traditional Chinese Medicine, Henan University of Traditional Chinese Medicine, Zhengzhou, China; ^3^ Department of Integrated Traditional Chinese and Western Medicine, The First Affiliated Hospital of Zhengzhou University, Zhengzhou, China

**Keywords:** immune microenvironment, immunotherapy, HCC, NK cell therapy, CAR-T therapy

## Abstract

Hepatocellular carcinoma (HCC) remains a leading cause of cancer-related mortality globally. The tumor microenvironment (TME) plays a pivotal role in HCC progression, characterized by dynamic interactions between stromal components, immune cells, and tumor cells. Key immune players, including tumor-associated macrophages (TAMs), tumor-infiltrating lymphocytes (TILs), cytotoxic T lymphocytes (CTLs), regulatory T cells (Tregs), MDSCs, dendritic cells (DCs), and natural killer (NK) cells, contribute to immune evasion and tumor progression. Recent advances in immunotherapy, such as immune checkpoint inhibitors (ICIs), cancer vaccines, adoptive cell therapy (ACT), and combination therapies, have shown promise in enhancing anti-tumor responses. Dual ICI combinations, ICIs with molecular targeted drugs, and integration with local treatments or radiotherapy have demonstrated improved outcomes in HCC patients. This review highlights the evolving understanding of the immune microenvironment and the therapeutic potential of immunotherapeutic strategies in HCC management.

## 1 Introduction

HCC constitutes 80-90% of Liver cancer cases, alongside intrahepatic cholangiocarcinoma and mixed-type carcinoma (1, 2). The tumor microenvironment (TME), a key driver of cancer progression, dynamically evolves from premalignant stages to advanced tumor development (3, 4). This evolution is characterized by the transition from a premalignant microenvironment to a tumor-promoting microenvironment, both of which adapt as the tumor progresses (5).

The immune microenvironment, comprising TILs, CTLs, Tregs, TAMs, and MDSCs, plays a pivotal role in the development and progression of HCC (6). HCC is frequently asymptomatic in its early stages, leading to the majority of cases being diagnosed at advanced phases, which significantly limits surgical intervention. Traditional therapeutic approaches often exhibit limited efficacy and are associated with high rates of recurrence. In contrast, immunotherapy has emerged as a transformative strategy, leveraging advances in immunobiology to enhance anti-tumor immune responses. This review summarizes the roles of immune cells within the TME and recent advancements in immunotherapy for HCC.

## 2 Immune microenvironment in HCC

### 2.1 TAMs

TAMs exhibit M1 and M2 phenotypes, with M2 promoting immune evasion and tumor progression. Glycolytic pathway inhibition suppresses M2 polarization (7). Receptor-interacting serine/threonine-protein kinase 3 (RIPK3)-deficient TAMs enhance M2 polarization via fatty acid oxidation, whereas decitabine modulates metabolism to boost anti-tumor immunity (8). TAMs drive HCC invasion, metastasis, and recurrence, correlating with poor prognosis (9). Hypoxia induces IL-1β release via TLR4/TIR-TRIF/NF-κB, promoting epithelial-mesenchymal transition (EMT) (10, 11). TAMs produce inflammatory cytokines, including TNF-α, IL-β, IL-6, and IL-23. They also expand Th17 cells and suppress immunity by upregulating PD-1, CTLA-4, and GITR (12–15). TGF-β upregulates TIM-3 on TAMs, enhancing tumor growth via NF-κB/IL-6 (16). Mitochondrial fission in HCC activates TLR9/NF-κB, increasing CD163^+^ TAM infiltration and CCL2, linked to poor prognosis (17). Furthermore, TAMs interact with cancer-associated fibroblasts (CAFs) via cytokines such as TGF-β and stromal cell-derived factor-1 (SDF-1), promoting M2 polarization, fibrosis, and immunosuppression, enhancing tumorigenesis and immune evasion (18).

### 2.2 TILs

TILs, comprising T, B, and NK cells, are crucial in antitumor immunity (19–22). FOXP3^+^ regulatory T cells (Tregs) act as tumor suppressors, while immunohistochemical studies demonstrate that immune cells regulate both antitumor responses and tumor-promoting conditions within the TME (23, 24). In HCC, the role of TILs in immune surveillance varies, with the balance between regulatory and cytotoxic T cells significantly influencing tumor progression (25). TILs are emerging as potential prognostic biomarkers in HCC, although findings on the impact of FOXP3^+^ and CD8^+^ T cells on prognosis remain conflicting (26–29).

### 2.3 CTLs

CTLs, guided by CD4^+^ T helper (Th) cells, target abnormal cells and initiate cytotoxic responses to eliminate tumors. Activated DCs support CTL function by providing CXCL16 and IL-15, which promote CTL accumulation and survival (30, 31). Despite immune suppression via hypoxia, metabolic competition, insufficient CD4^+^ T cells, and high expression of regulatory molecules (e.g., VEGF, CXCL17, IL-10, IDO), the presence of CD8^+^ CTLs in HCC correlates with improved survival (32–37). However, Fas/FasL expression in CD8^+^ T cells and endothelial cells, induced by VEGF-A and PGE2, accelerates CD8^+^ turnover, diminishing antitumor response (32, 38, 39). Additionally, CTLs are further suppressed by IL-2 and indoleamine 2,3-dioxygenase (IDO) secreted by CD14+ DCs (40).

### 2.4 Tregs

Tregs, marked by CD25 and FOXP3, maintain immune tolerance but promote tumor progression in the TME. They infiltrate cancers like melanoma, suppressing anti-tumor responses via CCL6/CCL20 and TCR/IL-10/TGF-β pathways (41, 42). In HCC, Tregs are elevated in tumor tissues and blood, with increased CD4^+^CD25^+^ cells (43, 44). LncRNAs and pro-inflammatory signals enhance Treg differentiation; lnc-Epidermal growth factor receptor (EGFR) overexpression inhibits ubiquitination via AP-1/NFAT1, aiding immune suppression (45). Tregs are key immunotherapy targets, with sorafenib reducing Tregs by blocking TGF-β (46). Various microRNAs, including miR-150-5p and miR-142-3p, are implicated in immunosuppression mediated by Treg cell-derived extracellular vesicles, which foster a tolerogenic state in dendritic cells (47).

### 2.5 Myeloid-derived suppressor cells

MDSCs exhibit immunosuppressive functions by inhibiting T cell activation, inducing T cell anergy, suppressing NK cell cytotoxicity, and polarizing macrophages towards a pro-tumor phenotype (48). MDSCs are broadly categorized into two subsets: monocytic (M-MDSCs) and polymorphonuclear (PMN-MDSCs). Under physiological conditions, MDSCs differentiate into macrophages, granulocytes, and dendritic cells; however, during inflammation or tumorigenesis, they undergo expansion and transform into immune-suppressive TAMs (49). Within the TME, MDSCs exert their immunosuppressive effects by depleting cysteine, upregulating inducible nitric oxide synthase (iNOS) and arginase-1 (ARG-1), impairing T cell function, promoting regulatory T cell (Treg) expansion, and generating reactive oxygen species (ROS) to inhibit NK cell activity (50). Notably, KRAS mutations in M-MDSCs suppress interferon regulatory factor 2 (IRF2), leading to the release of CXCL3, which recruits additional MDSCs and inhibits cytotoxic T cells (51). Clinical studies have demonstrated that elevated M-MDSC populations in HCC patients are closely associated with the induction of CD4+CD25+Foxp3+ Tregs and the suppression of NK cell activity through NKp30-dependent cell contact (52, 53). Furthermore, CCRK expression in tumors promotes MDSC accumulation, with high CCRK/IL-6/CD11b/CD33 levels linked to poor prognosis (54). Preclinical HCC models show CCRK signaling recruits MDSCs to establish an immune-suppressive TME, reversible by targeting CCRK or IL-6 (55). Besides, Treg-derived exosomes amplify the immunosuppressive capacity of MDSCs (56).

### 2.6 Dendritic cells

DCs is recognized as the most potent antigen-presenting cells, predominantly exist in an immature state *in vivo*, where they exhibit exceptional efficiency in antigen uptake. They activate T lymphocytes for anti-tumor responses, stimulate B cell maturation, and activate Th and NK cells. DCs are classified into cDC1 (myeloid-derived) and cDC2 (lymphoid-derived) subtypes. They present antigens via MHC-I/II pathways and induce co-stimulatory signals (e.g., B7/CD28) to activate CTLs for anti-tumor immunity (57). Antigen delivery by DCs may involve exosome vesicles (58). In HCC, LAMP3^+^ DCs, expressing CD80/83 and CCR7, migrate to liver lymph nodes, interacting with Tregs via CD86-CD28 and exhausted T cells via CD86-CTLA4 (5). HCC patients show reduced circulating pDCs and cDCs, with decreased co-stimulatory molecule expression, negatively correlating with IL-10 levels (59, 60). CD303^+^ pDCs in HCC tumors co-localize with type 1 Tregs and correlate with poor prognosis (61, 62).

### 2.7 Natural killer cells

NK cells, innate lymphocytes, mediate tumor cell killing via perforin/granzyme exocytosis, FASL/RAILR binding, and IFN-γ/TNFα secretion. Classified into CD56dim (mature, cytotoxic, blood-dominant) and CD56bright (immature, immunomodulatory, lymphoid organ-resident) subsets (63). In the TME, NK infiltration is limited, with suppressed function due to altered chemokine profiles, fructose-1,6-bisphosphatase-induced glycolysis suppression, and adenosine-mediated maturation inhibition (64, 65). High NK and CD8^+^ T cell presence correlates with favorable prognosis and tumor apoptosis in early HCC (66). However, in stage III HCC (67), CD56dim NK frequency declines, while immunoregulatory NKs expand, with tumor-infiltrating CD56dim NKs expressing PD-1/NKG2A, linked to poor prognosis (68, 69). Sorafenib enhances NK activity via macrophage-derived cytokines (IL-12, IL-18, IL-1β), suggesting NK activation contributes to its anti-tumor efficacy (70, 71).

### 2.8 Tumor-associated neutrophils

**Figure 1:**
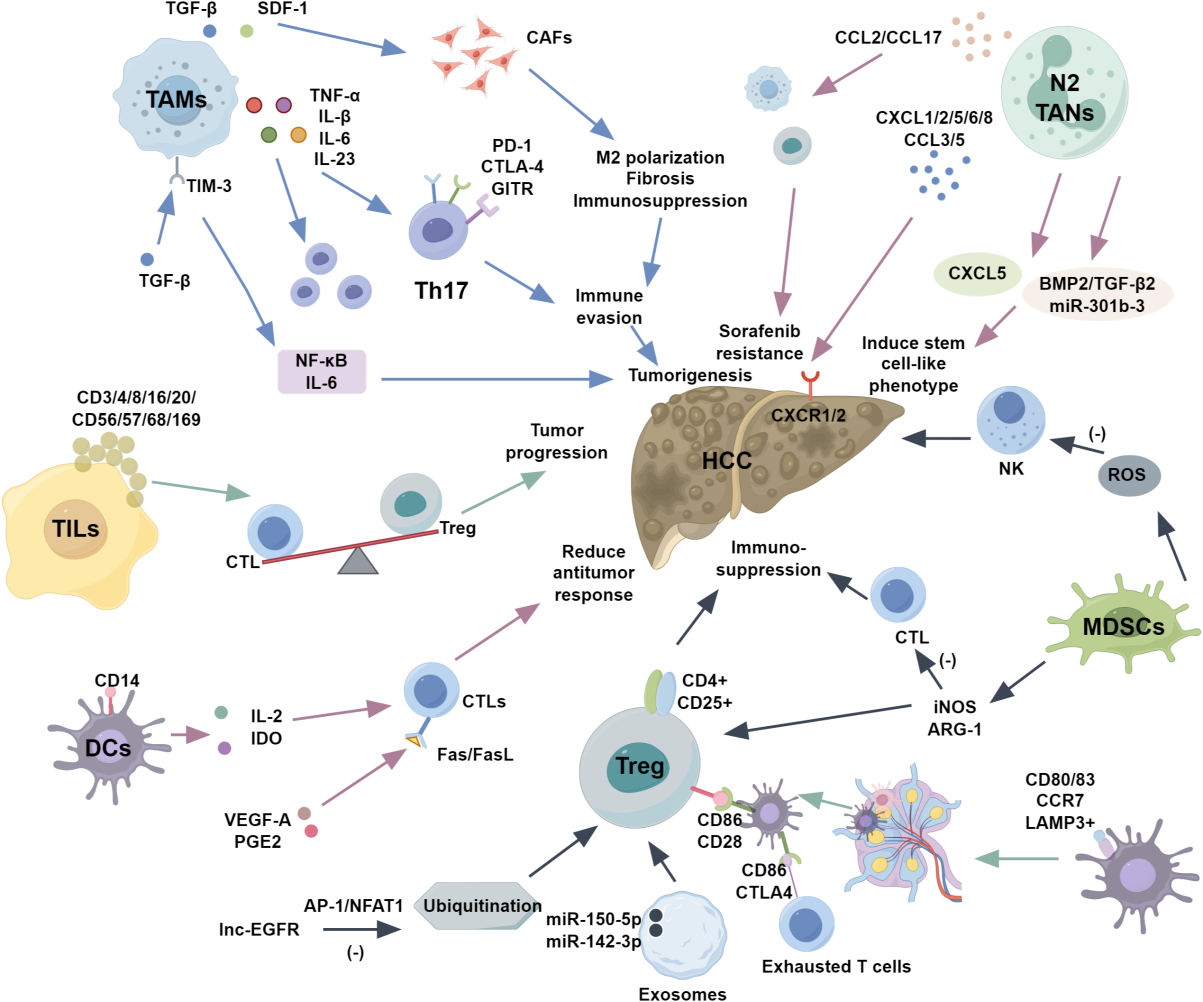
The role of immune cells in HCC.

TANs are guided into tumors by chemokines (CXCL1/2/5/6/8, CCL3/5) binding to CXCR1/2 receptors (72). TANs exhibit dual roles: promoting tumor growth via angiogenesis, ECM remodeling, and immunosuppression, or mediating anti-tumor responses through ROS, RNS, and direct cytotoxicity. Polarization to N1 (anti-tumor, IFNβ/IL-1β-driven) or N2 (pro-tumor, TGF-β/IL-6/7/8-driven) phenotypes influences tumor dynamics (73). The neutrophil-lymphocyte ratio is a prognostic marker in liver cancer (74). Neutrophil-tumor cell clusters enhance metastasis via upregulated cell cycle and DNA replication genes (75). N2 TANs induce stem cell-like phenotypes in HCC through BMP2/TGF-β2/miR-301b-3p signaling and CXCL5 feedback loops (76, 77). They recruit macrophages/Tregs via CCL2/CCL17, inducing sorafenib resistance (78), and form NETs, promoting HCC progression (79) (**Figure 1**).

## 3 Immunotherapy

### 3.1 Immune checkpoint inhibitors

#### 3.1.1 PD-1/PD-L1 inhibitors

The PD-1/PD-L1 axis, expressed on immune and tumor cells respectively, suppresses immune activation and cytotoxic function, particularly in liver malignancies (80). Clinical trials demonstrate PD-1 inhibitors’ efficacy: nivolumab (CheckMate-040) showed 23% ORR and 28.6-month median OS in sorafenib-naïve HCC (81), while pembrolizumab (KEYNOTE-240) improved survival versus placebo (82). Camrelizumab achieved 14.7% ORR and 14.2-month median OS in Chinese HCC patients (83). with comparable outcomes among PD-1 inhibitors (81, 83–85). PD-L1 inhibitors show promise, with atezolizumab plus bevacizumab (IMbrave 150) demonstrating superior 12-month survival versus sorafenib in unresectable HCC (86).

#### 3.1.2 CTLA-4 inhibitors

CTLA-4, a homolog of CD28 expressed on activated CD4^+^ and CD8^+^ T lymphocytes, competes with CD28 for binding to B7 ligands, thus inhibiting T lymphocyte activation (87). CTLA-4 inhibitors relieve this inhibitory signal, activating specific effector T cells to induce or enhance anti-tumor immune responses. Ipilimumab is currently the only approved CTLA-4 inhibitor. A study of 21 HCC patients treated with ipilimumab showed a partial response rate (PR) of 17.6% and a disease control rate (DCR) of 76.4%, demonstrating its significant therapeutic effect (88).

#### 3.1.3 Glucocorticoid-induced tumor necrosis factor receptor agonists

GITR agonists enhance anti-tumor immune responses through co-stimulation, making them a novel target in cancer immunotherapy. Preclinical studies have shown that GITR agonists promote the activation of CD8^+^ and CD4^+^ effector T cells while inhibiting the activity of tumor-infiltrating T cells. A phase I clinical trial of the GITR agonist monoclonal antibody TRx518 demonstrated promising safety and immune effects in patients with advanced cancer (NCT01239134) (89).

### 3.2 Cancer vaccines

Cancer vaccines are therapeutic vaccines targeting tumor-associated antigens, stimulating the body’s immune response to target and kill tumor cells. The main types of cancer vaccines include peptide vaccines, DC vaccines, and oncolytic virus vaccines.

#### 3.2.1 Peptide vaccines

Tumor peptide vaccines, utilizing surface antigens or intracellular peptides, activate T cell-mediated anti-tumor immunity (90–92). Glypican-3, overexpressed in HCC, demonstrated clinical efficacy, with Sawada et al. (93) showing reduced recurrence and improved survival when combined with surgery versus surgery alone. Multi-drug resistance protein 3 vaccines also enhanced survival in advanced HCC (94, 95). Glypican-3 trials induced CTL responses in 30/33 patients, with partial/stable disease and improved OSR objective survival rates (OSR) (96, 97). Phase II studies confirmed reduced post-surgical recurrence (93). Alpha-fetoprotein (AFP) vaccines activated HCC immunity (98), with hAFP-DCs enhancing anti-tumor effects in mice (99). AFP-derived peptides (AFP357,403) showed safety and efficacy, including complete remission (100), while AFP158-specific TCR genes enabled T cells to target HCC cells (101).

#### 3.2.2 DC vaccines

Dendritic cells (DCs), as highly potent antigen-presenting cells, can be effectively sensitized using tumor antigen extracts to elicit robust and specific immune responses. Accumulating evidence demonstrates that DC-based vaccines significantly enhance the proliferation and activation of CD8+ T lymphocytes while elevating serum interferon-gamma (IFN-γ) levels in patients with hepatocellular carcinoma (HCC), thereby contributing to improved overall survival (OS) (102). Meta-analyses confirm DC immunotherapy as an effective adjuvant for HCC, boosting antitumor immunity, survival rates, and reducing recurrence, with good safety (103). Notably, MIZUKOSHI et al. (104) provided compelling evidence that the combination of transcatheter arterial embolization (TAE) with DC infusion significantly amplifies tumor-specific T lymphocyte responses and enhances antitumor immunity compared to TAE monotherapy, highlighting the synergistic potential of this combined therapeutic approach. Post-surgery, RFA, TACE, or PEI followed by DC immunotherapy reduced recurrence risk and enhanced tumor-specific immunity (105). Combining DC vaccines or CIK with conventional treatments improved prognosis, OSR, and reduced recurrence (106). DC monotherapy significantly improved 1-year OSR, demonstrating its clinical utility as an HCC adjuvant therapy.

#### 3.2.3 Oncolytic virus vaccines

Oncolytic viruses proliferate only within tumor cells, causing tumor cell lysis, and exert dual effects by specifically killing tumor cells and activating the body’s antitumor immune response. JX-594, an oncolytic vaccinia virus, has been shown to have therapeutic effects in liver cancer. Heo et al. (107) injected the JX-594 virus vaccine into liver cancer tissue, confirming its therapeutic effect and demonstrating early efficacy. Additionally, Pexa-Vec, another oncolytic virus, has garnered widespread attention in HCC treatment. A clinical trial combining Pexa-Vec with sorafenib for advanced HCC patients showed that the combination treatment slowed disease progression and prolonged survival in patients with advanced HCC (108).

### 3.3 Cytokines

Cytokines, including IFN, IL, colony-stimulating factors, and TNF are immune cell-secreted signaling molecules. IFN exhibits antiviral, immune-regulating, anti-angiogenic, and pro-apoptotic effects, crucial in tumor treatment. Lee et al. (109) found PEGylated IFN reduced recurrence in post-surgery liver cancer patients. Bertelli et al. (110) showed IL-2 prolonged survival in inoperable HCC patients, highlighting cytokines’ role in liver cancer therapy.

## 4 Adoptive cell therapy

### 4.1 CAR-T therapy

CAR-T therapy involves genetically modifying T lymphocytes to express receptors targeting tumor antigens, which recognize specific ligands to exert antitumor effects (111). Target antigens, overexpressed in tumors but minimally in normal tissues, are crucial for efficacy. Common targets in liver cancer include GPC3, AFP, hepatocyte growth factor receptor, and MUC1. GPC3 CAR-T delayed disease progression in HCC with good safety (112). GPC3-CAR T cells showed tumor-clearing effects in HCC patient-derived xenograft models (113). A phase I trial (NCT02395250, NCT03146234) reported 2 of 13 advanced HCC patients achieved PR, supporting CAR-GPC3 T cell therapy’s safety and efficacy (114).

### 4.2 CIK therapy

Previous clinical studies have shown that CIK cell therapy enhances immune function, clears residual tumor foci, and reduces tumor recurrence. Currently, DC and CIK cells are co-cultured to form DC-CIK cells. By presenting tumor antigens to CIK cells, DCs make the immune response more specific, enhancing CIK cell-mediated tumor cell killing. Wang et al. (115) showed that after co-culturing GPC3-transfected DCs with CIK cells, the differentiation and IFN-γ secretion of CIK cells were promoted, inducing a specific killing effect against GPC3-expressing liver cancer cells. DC-CIK cell immunotherapy has broad application prospects in liver cancer treatment.

### 4.3 TCR-T therapy

TCR-T therapy targets tumor antigen peptides presented by the major histocompatibility complex (MHC), with its antitumor activity primarily depending on CD8^+^ T lymphocytes. As the hepatitis B virus (HBV) genetic fragments can integrate into the chromosomes of liver cells during chronic infection, TCR-T therapy can target HBV antigens in HCC cells, making TCR-T therapy particularly advantageous in HBV-related HCC (116).

### 4.4 NK cell therapy

NK cells, comprising 30%-50% of intrahepatic lymphocytes, are crucial in immune defense against viral infections and tumor development. Dysfunction in NK cells, including reduced numbers and functional defects, facilitates tumor immune evasion, contributing to HCC (117). Allogeneic NK cell immunotherapy is increasingly used in clinical trials. Combining irreversible electroporation with NK cell therapy significantly improved median OS in stage IV HCC patients (NCT03008343) (118). Another trial (NCT01147380) infused donor-derived activated NK cells into liver transplant recipients, showing safety and feasibility in preventing metastasis and recurrence (119). Genetic modification of NK cells is also explored to enhance specificity and efficacy.

## 5 Combination therapy

### 5.1 Dual ICI combination

Dual ICI combination therapy demonstrates synergistic effects, enhancing response rates and efficacy. Studies on PD-1/PD-L1 and CTLA-4 inhibitors in melanoma have informed HCC trials. In CheckMate 040, nivolumab and ipilimumab in sorafenib-treated HCC patients yielded an ORR of 34%, DCR of 51.2%, and 60-month survival of 29% (120). The CheckMate 9DW trial provided updated evidence supporting the significant OS benefits over sorafenib (121). The HIMALAYA trial established the combination of tremelimumab and durvalumab as an effective first-line therapy, achieving a median OS of 16.4 months compared to 13.8 months with sorafenib (122). GITR agonist antibodies with PD-1 inhibitors may overcome monotherapy resistance (89, 123). They enhance TIL responses in advanced HCC (124), which indicates improved anti-tumor efficacy.

### 5.2 ICI combined with molecular targeted drugs

Molecular targeted drugs inhibiting tumor angiogenesis can suppress the tumor’s immune-suppressive microenvironment and enhance immunotherapy sensitivity (125, 126). Combining immunotherapy with targeted treatment yields synergistic anti-tumor effects. Atezolizumab plus bevacizumab showed superior efficacy in advanced unresectable HCC than sorafenib alone (86). KEYNOTE-524 (127) and LEAP-002 trial (128) reported elevated ORR or OS in advanced HCC. These findings highlight the efficacy of ICI combined with targeted drugs, overcoming monotherapy limitations.

### 5.3 ICI combined with local treatment

HCC treatments like ablation and interventional therapies release tumor antigens, induce necrosis, and activate immune cells (CD8^+^ T-cells, NK cells). Combined with immunotherapy, they enhance anti-tumor effects. A study showed ablation with tremelimumab increased CD8^+^ T-cells, with median OS of 12.3 months (129). TACE, a non-surgical HCC treatment, induces tumor necrosis. DC-CIK with TACE prolonged PFS than TACE alone, suggesting immunotherapy combined with local treatments benefits non-surgical HCC patients (130).

### 5.4 ICI combined with radiotherapy

Combining 131I-Metoxymab, a radioactive fragment targeting HCC-associated CD147, with radiofrequency ablation (RFA) significantly lowered 1- and 2-year recurrence rates compared to RFA alone (131). This finding highlights the therapeutic potential of McAb-mediated radiotherapy through dual cytotoxic mechanisms in liver cancer treatment. In HCC murine models, combined Radiotherapy and anti-PD-L1 therapy enhanced survival and tumor control versus monotherapies, with Radiotherapy -induced PD-L1 upregulation via IFN-gamma/STAT3 potentially boosting immunotherapy (132). A matched analysis showed Radiotherapy addition to anti-PD-1 and anti-angiogenic therapy improved ORR, OS, and PFS (133) (**Table 1**).

**Table 1:** The immunotherapies in HCC.

Category	Subcategory	Details
Immune checkpoint inhibitors (ICI)	PD-1/PD-L1 Inhibitors	PD-1/PD-L1 axis suppresses immune function in HCC. Trials: Nivolumab (23% ORR), Pembrolizumab (improved survival), Camrelizumab (14.7% ORR).
	CTLA-4 Inhibitors	CTLA-4 inhibits T cell activation. Ipilimumab shows 17.6% PR and 76.4% DCR in HCC.
	GITR Agonists	Enhance T cell activation; TRx518 shows safety and immune effects in HCC.
Cancer vaccines	Peptide Vaccines	GPC3, AFP vaccines improve survival and recurrence rates in HCC.
	DC Vaccines	DC vaccines enhance CD8^+^ T cells, improve OS in HCC.
	Oncolytic Virus Vaccines	JX-594 and Pexa-Vec show promising results in HCC.
Cytokines	Interferons, ILs, TNFs	IFN, IL-2, and TNF show potential in HCC treatment.
Adoptive cell therapy (ACT)	CAR-T Therapy	GPC3-targeted CAR-T shows efficacy in HCC.
	CIK Therapy	DC-CIK therapy enhances immune response and tumor killing in HCC.
	TCR-T Therapy	Targets tumor peptides; useful in HBV-related HCC.
	NK Cell Therapy	NK cell immunotherapy shows potential in HCC.
Combination therapy	Dual ICI Combination	PD-1/PD-L1 + CTLA-4 combo improves anti-tumor efficacy.
	ICI + Molecular Targeted Drugs	Atezolizumab + bevacizumab and pembrolizumab + lenvatinib show improved outcomes.
	ICI + Local Treatment	Immunotherapy with ablation, TACE improves progression-free survival.
	ICI + Radiotherapy	Radiotherapy combined with anti-PD-L1 enhances survival and tumor control.

## 6 Conclusion

The immune microenvironment plays a pivotal role in the progression of hepatocellular carcinoma, with immune cells both promoting tumor growth and suppressing anti-tumor responses. The intricate interplay between TAMs, TILs, and MDSCs shapes the immune landscape, influencing the effectiveness of therapies. Immunotherapy, particularly the use of immune checkpoint inhibitors, has revolutionized treatment strategies for HCC, offering new hope for patients with advanced disease. The combination of ICIs with molecular targeted drugs, local treatments, or radiotherapy holds significant promise for improving treatment outcomes. However, HCC immunotherapy faces limitations due to low response rates and resistance, driven by TME-induced immune suppression, T-cell exhaustion, and immunosuppressive cells. Combination therapies show variable efficacy and pose risks of immune-related adverse events, necessitating deeper insights into resistance mechanisms and predictive biomarkers.
